# Early clinical experience with the Carina robotic platform in urologic surgery

**DOI:** 10.1002/bco2.70050

**Published:** 2025-07-16

**Authors:** Hongkai Wang, Dalong Cao, Marcio Covas Moschovas, Junlong Wu, Beihe Wang, Kun Chang, Hui Wang, Yun Zhu, Bo Dai, Yao Zhu, Hailiang Zhang, Vipul R. Patel, Dingwei Ye

**Affiliations:** ^1^ Department of Urology Fudan University Shanghai Cancer Center Shanghai China; ^2^ Urologic Oncology AdventHealth Global Robotics Institute Florida USA; ^3^ Department of Anesthesiology Fudan University Shanghai Cancer Center Shanghai China; ^4^ Operating Room Fudan University Shanghai Cancer Center Shanghai China

**Keywords:** blood loss, clinical outcome, docking time, modular surgical robot, robot‐assisted partial nephrectomy, robot‐assisted radical prostatectomy, total surgery time, complication

## Abstract

**Objectives:**

To evaluate the feasibility and safety of a new modular robotic surgical platform ‐ Carina Platform (Ronovo Surgical, Shanghai, China) in prostate and renal surgeries.

**Methods:**

We performed 11 robot‐assisted radical prostatectomies (RARP) and 7 robot‐assisted partial nephrectomies (RAPN) using the novel Carina Platform at Fudan University Shanghai Cancer Center (Shanghai, China). The port placements, operating space setting, cart distances and patient positions for all surgeries were recommended according to the procedure cards developed by Ronovo Surgical. Outcomes include success rate (completion without conversion to laparoscopy or open surgery), docking time, total surgery time and safety evaluations such as estimated blood loss (EBL) and complications.

**Results:**

The age of the patients ranged from 37 to 73 years, and the BMI ranged from 19.9 to 29.1 kg/m^2^. All operations were successfully completed without conversion to laparoscopy or extra port placement. For RARP, the mean docking time was 6.8 ± 5.6 min; the mean total surgery time was 156.3 ± 27.8 min; and mean EBL was 67.3 ± 39.2 ml. For RAPN, the mean docking time was 8.9 ± 1.6 min; the mean total surgery time was 146.0 ± 38.0 min; and mean EBL was 47.1 ± 7.6 ml. The mean warm ischemia time was 23.9 ± 6.7 min. There were no severe intraoperative or postoperative complications in 1‐month follow‐up.

**Conclusions:**

This is the first clinical reporting outcomes of the Carina Platform in urologic procedures. The study provides evidence of feasibility and safety when performing RARP and RAPN with acceptable perioperative outcomes and minimal complications.

## 1 INTRODUCTION

The relentless advancement of surgical techniques and the rapid development of surgical instruments guided minimally invasive surgery to the prevailing surgical approach for urologic oncology surgeries.^1^, ^2^ Robotic‐assisted surgery (RAS) stands out for its superior manoeuvrability as well as precision in suturing and anastomosis, and it has found widespread application in the surgical treatment of urinary system tumours. The most widely adopted RAS system to date is the DaVinci platform (Intuitive Surgical, Sunnyvale, CA, USA), having been utilized in over 10 million procedures in the past two decades.^3^, ^4^ In recent years, many new robotic platforms for laparoscopic surgery have been developed. It is believed that the latest generation of surgical robots, with a variety of new technologies and features, has the potential to increase the availability of RAS and lower the overall costs.^5^

Among these new robots, modular robotic systems are of great interest. These include Senhance (Asensus Surgical, USA.),^6^ Versius (CMR Surgical, UK.),^7^ Hugo RAS (Medtronic, USA)^8^ and others. The initial surgical experience of the Hugo RAS system in urology was published in 2022, and the authors indicated that the system showed satisfactory safety and feasibility.^8^, ^9^ It is believed that such modular architecture can facilitate a broader range of surgical configurations for diverse procedures, and the independent patient carts offer the flexibility to adjust the working angle without altering the port configuration, which is particularly advantageous in multi‐quadrant surgeries.^9^, ^10^, ^11^, ^12^

In this context, the Carina Platform is a new modular robotic surgery platform developed by Ronovo Surgical in Shanghai, China. Carina features a modular architecture with independent patient carts with a small footprint, an immersive surgeon console and a set of essential instruments for RAS. The system supports both wristed instruments and straight‐stick instruments adapted from conventional laparoscopic instruments. This has the potential to lower per‐procedure cost when straight‐stick instruments are used in combination with wristed instruments. Carina also supports 3D endoscope with fluorescence imaging capability from Ronovo Surgical (currently under development) and mainstream 3D endoscopes from manufacturers like Olympus, Karl Storz, etc.

Therefore, with consideration for the current evolution of modular robotic platforms, we describe the first clinical experience in urologic surgeries using Carina.

## 2 MATERIALS AND METHODS

### 2.1 Data source and patient selection

All procedures were completed at Fudan University Shanghai Cancer Center (Shanghai, China) between July and December 2023. These included 11 Robotic‐Assisted Radical Prostatectomy (RARP) and 7 Robotic‐Assisted Partial Nephrectomy (RAPN). Institutional ethical approval was granted (IRB No. 2309281–15‐2310A) and patients were counselled preoperatively with informed consent. All members of the surgery team had undergone extensive training for the Carina Platform.

We included consecutive patients between 18 and 80 years of age who already planned to undergo laparoscopic prostatectomy or partial nephrectomy. All patients agreed to participate in this study and voluntarily signed the informed consent form and expressed willingness to cooperate in completing research follow‐up and related examinations.

### 2.2 Data collection

The outcomes we measured included success rate (rate of completion without conversion to laparoscopy or open surgery), console time (min), total surgery time (min) and docking time (min). Clinical pathological data of the patients were recorded: age, BMI, prostate volume, pre‐operation PSA, Gleason score, post‐operative PSA, urinary continence one month after surgery, tumour size, R.E.N.A.L score^13^ for kidney tumours, as well as pre‐operative and post‐operative creatinine.

Safety evaluation were done by assessing the incidence of severe complications (Clavien‐Dindo complication grade 3 or above),^14^ intraoperative EBL, intraoperative blood transfusion rate and postoperative complications rate. Safety results were collected from the start of each surgery until 30 days post‐operation. Parametric data are presented as mean ± standard deviation and non‐parametric data are presented as median (range).

### 2.3 Carina platform

**FIGURE 1:**
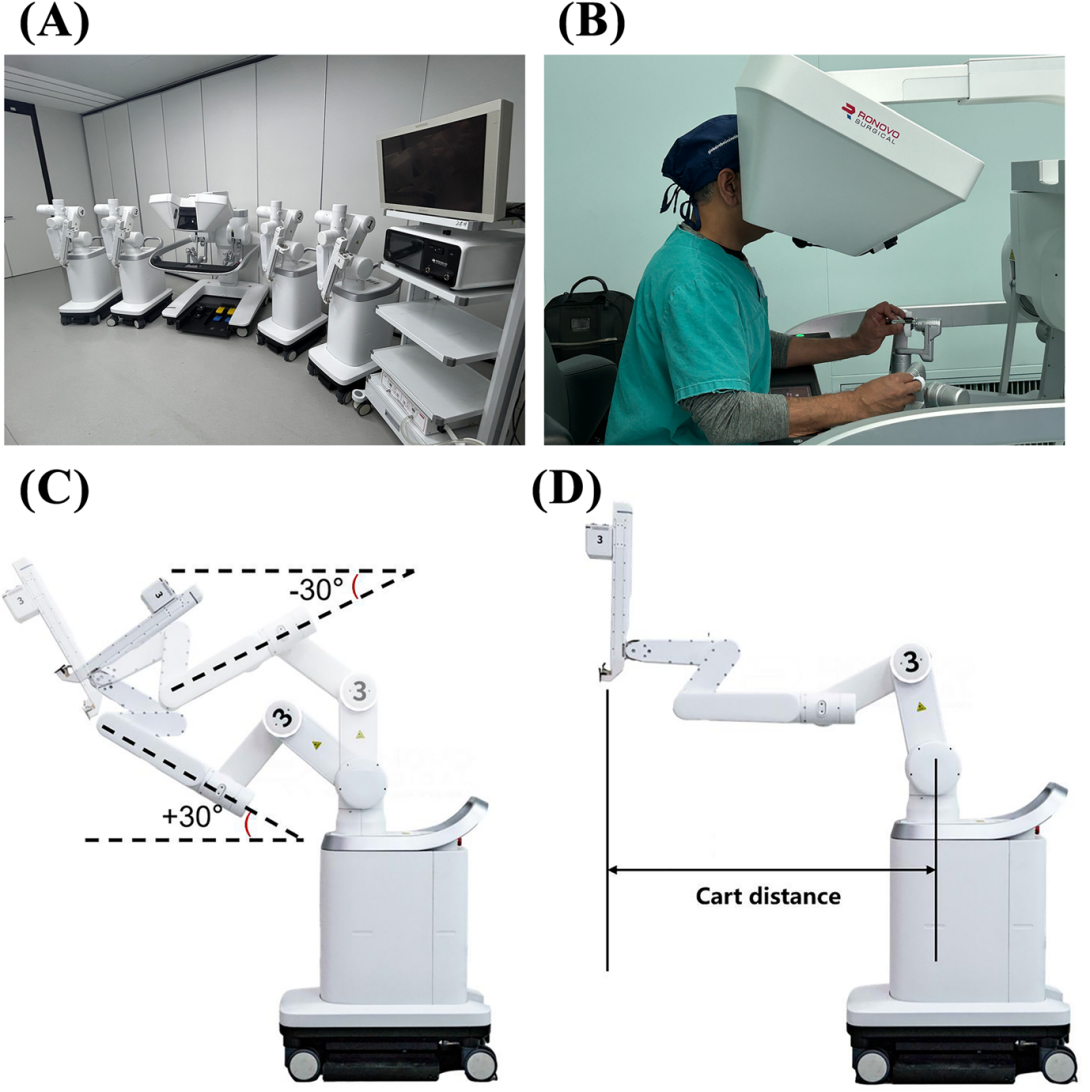
**(**a) The Carina platform with four patient carts, a surgeon console and an integration hub. (b) The immersive console provides the surgical view in stereoscopic 3D visualization. (c) Operation space setting:‐30° and +30° position. (d) Cart distance.

The Carina Platform features a modular design with small and independent patient carts **(**Figure 1a **)**. The surgical team can flexibly set up Carina with three or four carts, depending on the needs of the procedure. The surgeon sits ergonomically at the immersive console to see the surgical view in stereoscopic 3D visualization (Figure 1b). The integration hub serves as an intermediate unit between the surgeon console and patient carts, connecting the endoscope, the energy platform, the monitor and other equipment. It undertakes the functions of information transmission, peripherals connection and system control during operation. Each arm has different operation space settings (Figure 1c) and cart distance (Figure 1d) to achieve an optimal trocar placement and instrument movement during the surgery. Both wristed instruments and straight‐stick instruments can be used in combination, depending on the surgeon's surgical needs and preference.

To drape the patient cart, the robotic arm portion can be deployed to a draping configuration in which the drapes can be installed easily. Placing the patient carts around the surgical table, which is done according to the procedure card provided by Ronovo Surgical, involves three steps: 1) automatically deploy each robotic arm using buttons to a configuration where the cart distance is determined appropriate for the procedure, 2) place each patient cart at the location illustrated on the procedure card with the robotic arm portion pointing at the corresponding port site and 3) lock the patient cart in position. To stow away the patient cart, the robotic arm can be folded into a compact configuration using buttons on the arm, and then the cart can be rolled to a corner of the operating room or into the hallway.

The port placement, operation space setting, cart distance and patient position for RARP and RAPN were recommended based on the corresponding procedure cards. Prior to the present case series, the various settings detailed by the procedure cards were developed using computer simulations, and later verified and optimized through a multitude of preclinical porcine and cadaver labs.

### 2.4 Radical prostatectomy (RARP)

**FIGURE 2:**
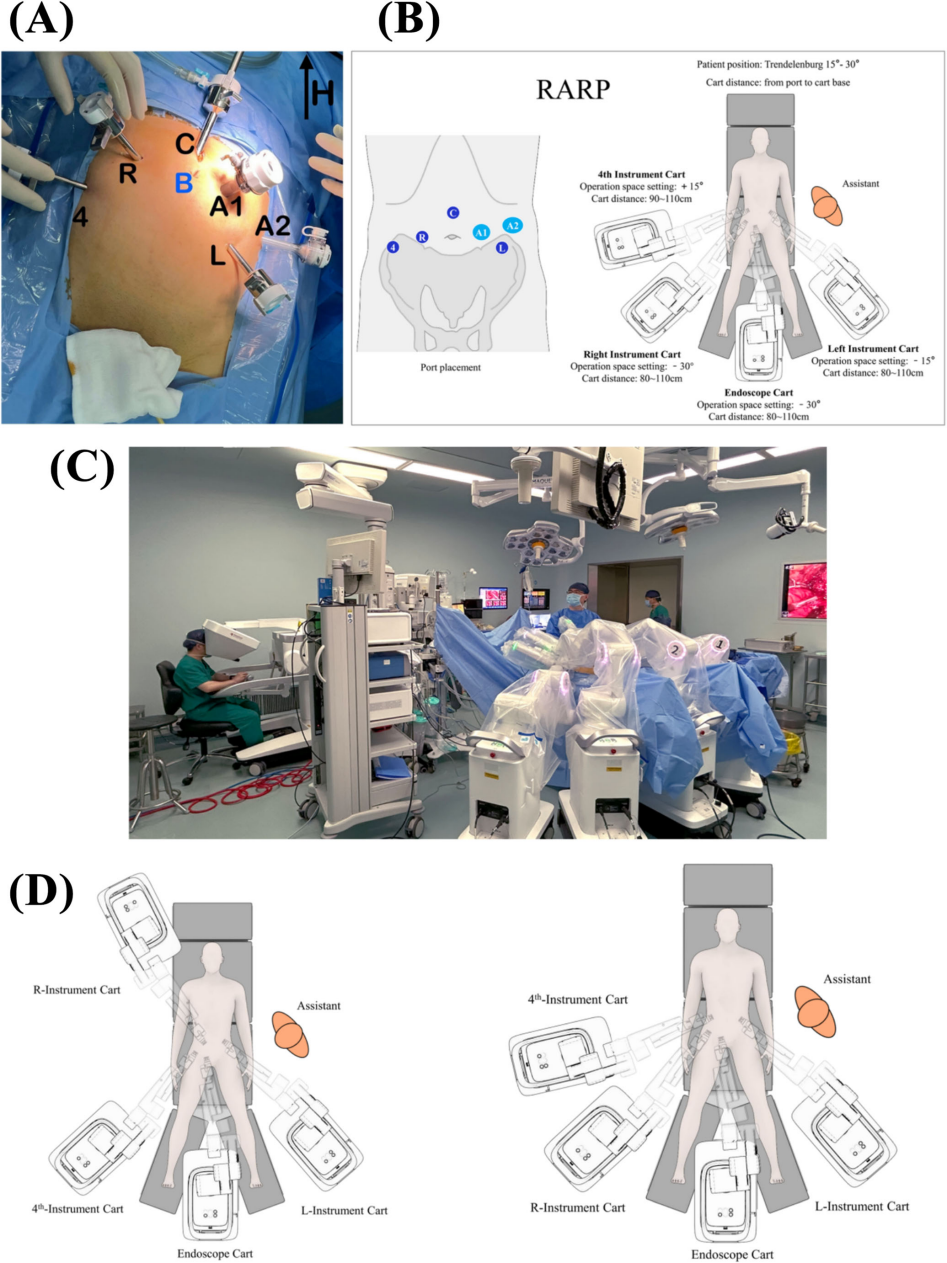
(a) Example of port placements for typical RARP performed with Carina. (b) Recommended port placements and patient cart configurations for prostatectomy. (c) Docked setup of Carina system for typical RARP. (d) Right instrument cart could be to the right of the head or beside the right leg for RARP.

For RARP, patients were placed in a 15‐to‐30‐degree Trendelenburg position with their legs separated by about 30 degrees prior to docking Carina. The port placements, operation space settings and cart distances were configured as prescribed by Ronovo Surgical (Figure 2a‐b). The camera port was placed in the supraumbilical position. Typical Carina system setup for RARP is shown in Figure 2c. Additionally, two patient cart configurations were utilized (Figure 2d). A sample of surgery video is uploaded (Supplementary material, Video S1 **)**.

In this case series, we primarily used wristed instruments on Carina, including needle driver, monopolar scissors, bipolar graspers and fenestrated forceps. The step‐by‐step RARP followed our previously described technique.^15^, ^16^, ^17^, ^18^

### 2.5 Partial nephrectomy (RAPN)

**FIGURE 3:**
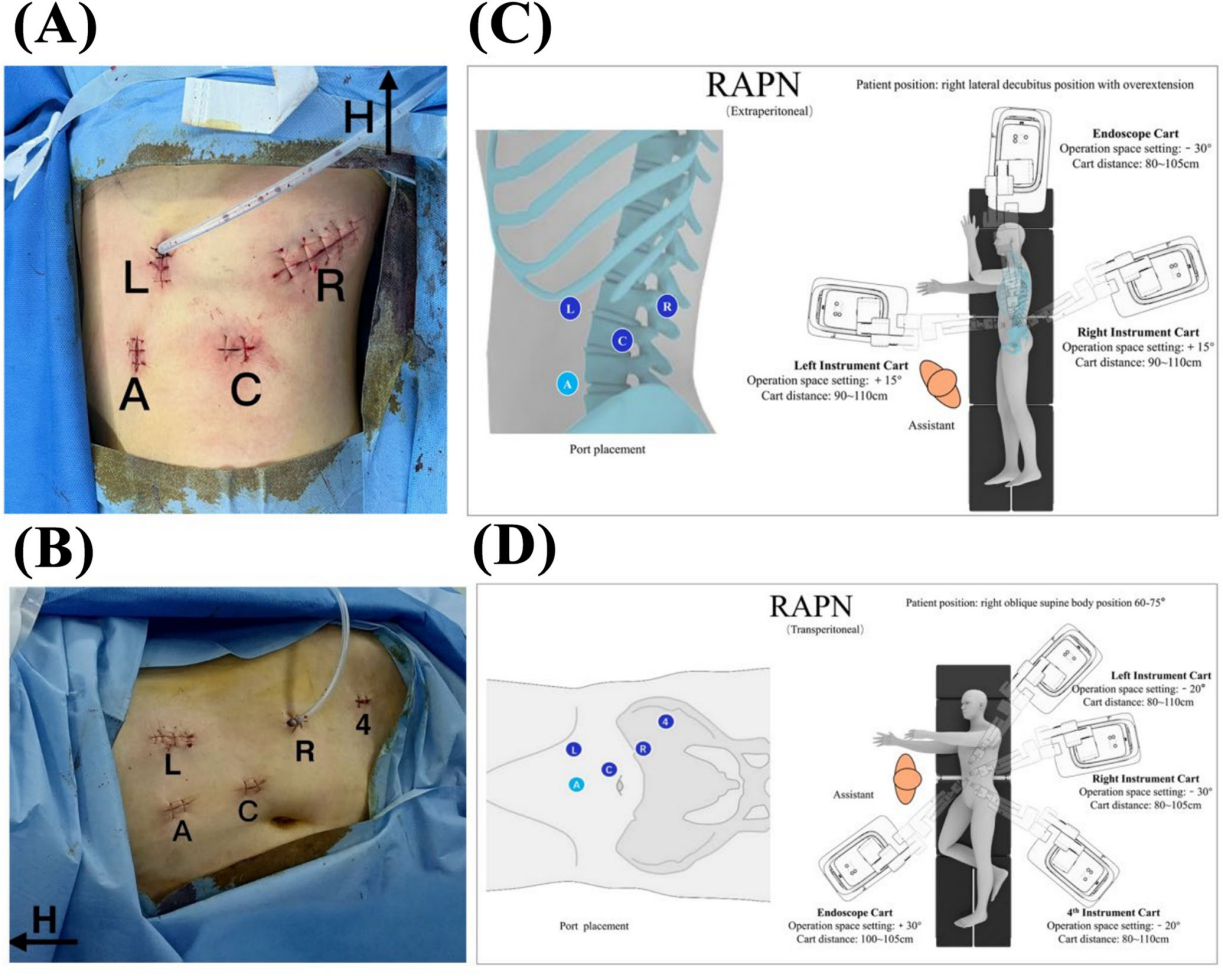
Demonstration of RAPN with Carina (left side as example): (a) port placements of extraperitoneal approach. (b) Port placements of transperitoneal approach. (c) Recommended port placements and patient cart configurations of extraperitoneal approach. (d) Recommended port placement and patient cart configurations of transperitoneal approach.

For RAPN, the port placements, operation space settings and patient cart positions for either extraperitoneal or transperitoneal approach are shown in Figure 3a‐d (left kidney tumour). During these surgeries, needle drivers, monopolar scissors, bipolar graspers and fenestrated forceps were used. Surgical procedures were performed with both extraperitoneal and transperitoneal approaches, as previously described.^19^, ^20^, ^21^

## 3 RESULTS

All operations were successfully completed with the Carina Platform without conversion to laparoscopy or extra port placement.

### 3.1 Radical prostatectomy (RARP)

Our case series covered representative prostatectomy variants, including transperitoneal prostatectomy, intra‐fascia nerve sparing radical prostatectomy using Vattikuti Institute prostatectomy technique^22^ and Dr. Vipul Patel's technique.^15^, ^16^, ^17^, ^18^ Four patients undergone pelvic lymph node dissection. The clinical data of the 11 patients are shown in Supplement Table S1. The median age of the patients was 67 years (59–73), the median BMI was 24.9 kg/m^2^ (21.2–29.3), the median prostate volume was 33.1 ml (8.1–77.5) and the median preoperative PSA was 11 ng/ml (0.007–68). Three patients had International Society of Urological Pathology (ISUP) grade 5 tumour on prostate biopsy, two had grade 4, one had grade 3, the rest were ≤ grade 2.

The mean system docking time was 6.8 ± 5.6 minutes. We observed the longest system docking time of 22 minutes for the first patient of the case series, which is understandable as this was the first human case ever performed with Carina and the surgical team was at the start of the clinical learning curve. However, docking time quickly reduced to 11 minutes for the next patient. The console time was 100.5 ± 24.8 minutes. The longest console time (144 minutes) was recorded in a procedure with higher BMI (29.3 kg/m^2^) patient who had a narrow pelvis. The total surgery time was 156.3 ± 27.8 minutes. The EBL was 67.3 ± 39.3 ml. No severe complications occurred, and the Foley catheter was usually removed 10–12 days after surgery. PSA was tested one month after surgery, and 10 patients achieved a PSA < 0.1 ng/ml. However, we observed failure to achieve a PSA < 0.2 for one patient, who was postoperatively diagnosed with oligo‐metastasis by PSMA‐PET/CT. Six patients used no more than one pad daily one‐month post‐surgery.

### 3.2 Partial nephrectomy (RAPN)

We included tumour resection in ventral and dorsal sides as well as upper, middle and lower pole of the kidney, performed with both extraperitoneal and transperitoneal approaches. Some cases were challenging, such as a case of solitary kidney and two cases with tumour in close proximity to the renal collecting system. The data of the 7 patients are shown in Supplement Table S2.

Mean docking time was 8.9 ± 1.6 minutes. Operation time and console time were 146.0 ± 38.0 and 62.6 ± 24.9 minutes, respectively. Warm ischemic time (WIT) was 23.9 ± 6.7 minutes. We observed the longest console time (102 minutes) and WIT (35 minutes) occurred in a patient with R.E.N.A.L. score of 10c. The EBL was 47.1 ± 7.6 ml. No severe intraoperative complications occurred. No blood transfusion was needed. Preoperative serum creatinine levels were comparable to postoperative levels 4 days after surgery: 65.1 ± 14.3 vs 67.7 ± 14.6 μmol/l. All 7 patients were discharged as expected, and no severe complications were found at 1‐month follow‐up.

## 4 DISCUSSION

Overall, we demonstrated the feasibility and safety of the Carina Platform in urological procedures. We performed 11 cases of RARP and 7 cases of RAPN using Carina, and all surgeries were successfully completed within a reasonable timeframe and without any severe complications. The postoperative follow‐up also showed satisfying results.

As clinical use of new surgical technologies is always challenging,^23^, ^24^ patient safety is of utmost importance. The surgical team had gone through extensive training with the clinical team at Ronovo Surgical prior to and in preparation for this case series. As confidence in the system performance grew, we began to perform more challenging cases and other variants of prostatectomies, and this progression was reflected in the console times, which rapidly reached competency level after only a few cases.

It is worthwhile to mention that the individual patient carts of Carina are hot‐swappable in the event of malfunction, allowing surgery to continue within minutes. Although we fortunately did not experience such a scenario during this case series, this backup capability gave us additional confidence regarding patient safety. It may seem time‐consuming to configure three or four patient carts in accordance with procedure cards, but the bedside team quickly realized that many steps can be done in parallel while the surgeon or physician assistant creates the ports for the patient. Docking times for both RARP and RAPN were reasonable and seem to stabilize after 5 cases.

Compared to the conventional robotic surgery system, Carina holds a key advantage in its independent patient carts, which can be flexibly configured to suit different workflows across multitudes of surgeries. Firstly, we were able to complete each procedure with a single docking of the patient carts. Neither post‐positioning measurements of cart locations nor intraoperative cart repositioning were needed. Secondly, we used two different cart layouts for RARPand both were feasible. Thirdly, with only 7 RAPN cases into the study, we were able to perform the cases transperitoneally or extraperitoneally with 3 or 4 patient carts, respectively.

The Carina patient carts are compact with a footprint of less than 0.3 square meters. In the RARP and RAPN procedures, 3 or 4 patient carts were placed around the operating table. We observed that with Carina fully deployed in the operating space, the physician assistant still had ample room to work and move about. When locked in place, the patient cart remained stable when leaned on or bumped against (by the physician assistant). When docked, the robotic arm is at the chest level of a typical physician assistant, leaving the space above Carina and over the operating table wide open, providing the bedside team with an unobstructed view of the operating room.

For RARP, we used the typical port placement on the conventional robotic surgery system. For RAPN,^25^ we slightly modified typical laparoscopic port placement by moving the scope port to the head side and the instrument ports away from the midaxillary line. This increases the distances between the ports to avoid potential collision among the robotic arms.

The high‐resolution camera on Carina provides immersive stereoscopic vision that improves depth perception.^26^ We paired the immersive 3D display on Carina with a 3D full HD endoscope from a local manufacturer for RARP cases and with a Karl Storz 3D HD endoscope for RAPN cases. The vision experience was adequate for RARP cases. The Karl Storz endoscope already used by the lead surgeon was the vision system of choice for RAPN cases, which also demonstrates the ability for Carina to leverage existing vision tower infrastructure in the OR to lower upfront cost of acquisition.

Instrument operation and monopolar/bipolar energy delivery were as expected. In all procedures, we primarily operated with wristed instruments that presented satisfactory traction and energy delivery to the tissues. The wristed instrument tip provides seven degrees of freedom inside the patient, permitting fine dissection and accurate suturing. Furthermore, the needle holder, which guides the suture needle through the tissue with minimal resistance, allows us to perform a tensionless anastomosis and easy knotting, especially in vesicourethral anastomosis when performing the van Velthoven running suture technique and posterior reconstruction.

In this clinical case series, all patients were followed up for a period of 30 days and have not reported any severe complications. With regard to functional outcomes, the rate of social continence (defined as the use of 0–1 pads) was 54.5% at one‐month post‐surgery, which is slightly lower than the rate of 59.5% reported in the literature.^27^ However, the postoperative evaluation was limited by the relatively brief duration of follow‐up which prevents direct comparison with findings in the literature, where the social continence rate was assessed after at least three months.^28^, ^29^ Therefore, an extended follow‐up period would be necessary for a comprehensive assessment.

Our study is not devoid of limitations. Firstly, the patient sample size is small, making it challenging to draw definitive conclusions. Additionally, the short‐term follow‐up and the lack of a comparison group constrain the analysis of the advantages of Carina over other robotic platforms. However, this is the first report of this technology application in humans, and we could perform our study with minimal complications and satisfactory perioperative outcomes.

## 5 CONCLUSIONS

Our study represents the first clinical experience to evaluate the feasibility and safety of the Carina Platform in urologic procedures, specifically RARP and RAPN. Our findings demonstrate that Carina can be successfully utilized to perform these complex urological procedures with acceptable perioperative outcomes and minimal complications. Further investigation and validation in larger cohorts are needed in the future.

## CONFLICT OF INTEREST STATEMENT

The authors declare that there is no conflict of interest regarding the publication of this paper.

## Supporting information


**Table S1:** Individual clinical characteristics of patients treated with robotic‐assisted radical prostatectomy using Carina.

**Table S2:** Individual clinical characteristics of patients treated with robotic‐assisted nephron sparing surgery performed with Carina.

**Video S1:** System Setup & RARP sample
