# Supplementary material for: Early clinical experience with the Carina robotic platform in urologic surgery
Source: BJUI Compass. 2025 Jul 16;6(7):e70050. doi: 10.1002/bco2.70050 (PMC12266805; doi:10.1002/bco2.70050)
Supplement: Supplementary file 1 — Table S1: Individual clinical characteristics of patients treated with robotic‐assisted radical prostatectomy using Carina. [file BCO2-6-e70050-s001.docx]

**Table 1 – Individual clinical characteristics of patients treated with robotic-assisted radical prostatectomy using Carina.**

| **Case No.** | **Age** | **BMI** | **Prostate Volume** | **Pre-Operative PSA** | **Gleason Score** | **Surgery type** | **Docking Time** | **Console Time** | **Total Surgery Time** | **Estimated Blood Loss** | **Hospital Stay** | **1-Month Post-Operative PSA** | **1-month Post-Operative pad use** |
| --- | --- | --- | --- | --- | --- | --- | --- | --- | --- | --- | --- | --- | --- |
|  | **(year)** | **(kg/m²)** | **(cm^3^)** | **(ng/ml)** |  |  | **(min)** | **(min)** | **(min)** | **(ml)** | **(day)** | **(ng/ml)** |  |
| **1** | 66 | 23.4 | 27.3 | 16 | 4+4 | RP | 22 | 140 | 195 | 50 | 6 | 0.072 | 3 pads |
| **2** | 63 | 24.1 | 36.7 | 8.8 | 4+3 | RP+PLND | 11 | 100 | 190 | 50 | 5 | 0.02 | 2 pads |
| **3^#^** | 71 | 29.1 | 8.1 | 0.007 | 4+5 | RP | 6 | 94 | 135 | 30 | 6 | 0.006 | 1pads |
| **4** | 73 | 26.9 | 26.1 | 10.7 | 3+4 | RP | 3 | 86 | 125 | 30 | 5 | 0.016 | 0pads |
| **5** | 60 | 22.3 | 20.1 | 9.8 | 3+4 | RP+NS | 3 | 94 | 138 | 30 | 6 | 0.008 | 1pads |
| **6** | 59 | 25.9 | 35.7 | 11 | 3+4 | RP+NS | 5 | 102 | 138 | 150 | 6 | 0.007 | 0pads |
| **7^#^** | 67 | 24.9 | 37.5 | 0.75 | 5+4 | RP+PLND | 3 | 116 | 175 | 100 | 6 | 0.006 | 2pads |
| **8** | 61 | 29.3 | 46.6 | 17 | 3+3 | RP+NS | 8 | 144 | 195 | 100 | 5 | 0.04 | 0pads |
| **9** | 70 | 21.2 | 77.5 | 25 | 3+4 | RP+NS | 5 | 82 | 150 | 50 | 6 | 0.022 | 1pads |
| **10** | 67 | 26 | 33.1 | 13 | 3+4 | RP+PLND | 5 | 60 | 155 | 100 | 7 | 0.596 | 2pads |
| **11** | 71 | 21.3 | 28.6 | 68 | 4+4 | RP+PLND | 4 | 88 | 123 | 50 | 6 | 0.072 | 2pads |

**^#^** Neoadjuvant androgen deprivation therapy (ADT) for 3 months.

RP：radical prostatectomy, PLND: pelvic lymph node dissection, NS: nerve sparing
