# Supplementary material for: Early clinical experience with the Carina robotic platform in urologic surgery
Source: BJUI Compass. 2025 Jul 16;6(7):e70050. doi: 10.1002/bco2.70050 (PMC12266805; doi:10.1002/bco2.70050)
Supplement: Supplementary file 2 — Table S2: Individual clinical characteristics of patients treated with robotic‐assisted nephron sparing surgery performed with Carina. [file BCO2-6-e70050-s003.docx]

**Table 2 – Individual clinical characteristics of patients treated with robotic-assisted nephron sparing surgery performed with Carina.**

| Case No. | Age (year) | BMI (kg/m²) | Tumor Maximum Diameter (cm) | R.E.N.A.L Score | Docking Time (min) | Console Time (min) | Total Surgery Time (min) | Estimated Blood Loss  (ml) | WIT (min) | Hospital Stay (day) | Pre-Surgery creatinine  (μmoI/L) | Post-Surgery creatinine  (μmoI/L) | Surgery approach |
| --- | --- | --- | --- | --- | --- | --- | --- | --- | --- | --- | --- | --- | --- |
| 1 | 37 | 26 | 1.8 | 4a | 11 | 60 | 144 | 30 | 29 | 6 | 86 | 85 | extraperitoneal |
| 2 | 42 | 20 | 4.8 | 10c | 10 | 102 | 224 | 50 | 35 | 9 | 44 | 52 | extraperitoneal |
| 3 | 58 | 24.2 | 1.1 | 6a | 10 | 48 | 158 | 50 | 16 | 8 | 75 | 87 | extraperitoneal |
| 4 | 47 | 19.9 | 3.7 | 7ab | 9 | 78 | 130 | 50 | 25 | 6 | 55 | 53 | transperitoneal |
| 5 | 60 | 26.1 | 1.2 | 6a | 7 | 78 | 133 | 50 | 25 | 5 | 60 | 63 | extraperitoneal |
| 6 | 42 | 26.7 | 1.7 | 4a | 7 | 42 | 128 | 50 | 18 | 6 | 61 | 59 | extraperitoneal |
| 7 | 52 | 26.2 | 2.1 | 4a | 8 | 30 | 105 | 50 | 19 | 5 | 75 | 75 | extraperitoneal |
